# Health Is Belonging: Lived Experiences during Recovery after Pancreaticoduodenectomy

**DOI:** 10.5402/2012/602323

**Published:** 2012-12-05

**Authors:** Thomas Andersson, Kristin Falk, Kristofer Bjerså, Anna Forsberg

**Affiliations:** ^1^Department of Surgery, Sahlgrenska University Hospital, 41345 Gothenburg, Sweden; ^2^Institute of Health and Care Sciences, Sahlgrenska Academy at University of Gothenburg, 40530 Gothenburg, Sweden; ^3^Department of Health Sciences, Faculty of Medicine, Lund University, Box 157, 22100 Lund, Sweden

## Abstract

The aim of this study was to explore the lived experience of the symptoms, health, and illness reported by patients recovering after pancreaticoduodenectomy ad modum Whipple due to pancreatic or periampullary cancer. Thirteen patients with pancreatic or periampullary cancer who underwent pancreaticoduodenectomy ad modum Whipple between 2006 and 2008 were interviewed during postoperative recovery. Data were analysed using the phenomenological-hermeneutic method. The structural analysis of patient experiences revealed that recovery after pancreaticoduodenectomy was described as recapturing everyday life, being healthy, and looking to the future. Participants experienced symptoms but did not dwell on them, instead they stated that their general health was good. They strived to regain their former precancer selves and be a part of as well as contribute to the social context. Overall, the participants' view of the future was positive, and improvement in their health generated further confidence and encouragement. This study suggests that persons recovering from pancreaticoduodenectomy ad modum Whipple due to a pancreatic or periampullary tumour experience health despite postoperative symptoms. They manage their symptoms by means of different strategies and express a positive view of the future. Nurses working with such patients should adopt a person-centred approach focusing on patient perspectives, participation, and possibilities.

## 1. Introduction

This paper reports an inside perspective of persons who underwent pancreatic cancer surgery. It adds new knowledge about the needs and preferences of this group of surgical patients and the nursing interventions required to promote postoperative recovery and health.

Pancreatic cancer is a relatively rare type of gastric cancer with high mortality. In 2008, there were 144,859 cases of pancreatic cancer in the world [1], of which approximately 37,680 were in the United States [2]. In Sweden, 1,250 cases of pancreatic and periampullary tumours were reported in 2010 [3]. Median survival following curative treatment by surgery and adjuvant therapy was 20.1–23.6 months, while median survival with palliative treatment was 5–9 months [4]. Five-year survival was less than 5% [5]. 

About 90% of all pancreatic tumours originate in the exocrine part of the pancreas and are usually localised to the pancreatic head (caput). These tumours generally manifest earlier than those in distal parts of the gland due to jaundice related to obstruction of the bile duct. Periampullary cancer includes tumours in ampulla vateri, distal bile duct, and duodenum. These are not clinically separate from pancreatic tumours but have a better prognosis [6]. Surgery is the only treatment that offers a cure for patients suffering from pancreatic adenocarcinoma [7].

Common surgical procedures for pancreatic cancer are pylorus preserving pancreaticoduodenectomy or pancreaticoduodenectomy (classic Whipple). There are no differences in mortality, morbidity, or long-term survival between pylorus preserving pancreaticoduodenectomy and classic Whipple in the treatment of pancreatic and periampullary cancer [8]. By clinical experience, hospital stay in Sweden after pancreatic surgery is two to three weeks. 

Several studies have reported four main symptoms of pancreatic cancer that are still present during recovery after pancreatic surgery [9–14]: weight loss, abdominal pain, lack of appetite and fatigue. In addition, Holly et al. [9] and Sun et al. [13] indicated sleep difficulties and reduced ability to perform everyday activities.

Fitzsimmons et al. [15] found that quality of life (QoL) among persons with pancreatic cancer was related to coping strategies and the level of experienced threat caused by symptoms, as opposed to the symptom burden itself. The main coping strategies for dealing with symptoms were defending/avoidance, blaming, rationalising, turning to others, and taking direct action. Several studies of surgically treated patients with pancreatic cancer have reported up to six months of postoperative QoL impairment [16–19]. However, at present, there is a lack of inductive qualitative studies that exclusively investigate the lived experience of patients who underwent pancreatic surgery.

In conclusion, previous research has been conducted with an outside perspective, based on the assumption that all patients treated by means of upper gastric surgery have a homogeneous experience of recovery. This paper builds on the view that every person is unique and that various forms of upper gastric cancer are experienced differently. Thus, there is a need for an inside perspective from persons who underwent surgery for pancreatic cancer. The aim of the present study was to explore the lived experience of the symptoms, health, and illness reported by patients recovering after pancreaticoduodenectomy ad modum Whipple due to pancreatic and periampullary cancers. 

## 2. Method

An inductive and exploratory study was conducted over a two-year period at the Sahlgrenska University hospital in Gothenburg. 

### 2.1. Participants

Patients were identified through clinical records by the first author. Seventeen potential participants who had undergone pancreaticoduodenectomy ad modum Whipple between 2006 and 2008 were contacted. Inclusion criteria were

- having undergone pancreaticoduodenectomy ad modum Whipple for a pancreatic or periampullary tumour,
- no experience of other major surgery or reoperation,
- no mental disorder,
- no alcohol or drug abuse,
- time since discharge not less than 30 days.

Written information about the study including an invitation to participate was sent to the seventeen patients. A week later, the author contacted them by phone, provided verbal information about the study, and asked if they would like to take part. Thirteen patients, nine women, and four men, with a mean age of 66.5 years (54–76 years), agreed to participate and were interviewed on one occasion. Four patients declined participation. Demographic details are presented in Table 1. 

### 2.2. Data Collection

Prior to the interviews, which were conducted by the first author, the participants gave their written consent. They were allowed to choose the location of the interview, six of which took place at the surgical unit and seven in the informants' own homes. Each interview lasted between 20 and 60 minutes and was audio taped and transcribed verbatim. The interviews began with an open question “*Can you please tell me about your experiences after discharge from hospital?*” followed by more specific questions; “*Can you please tell me about your everyday life and how it is affected by the operation?*” and “*What does health mean to you at this point in your post surgical rehabilitation*?”. The purpose of these follow-up questions was to obtain clarification and deepen understanding.

### 2.3. Data Analysis

A phenomenological-hermeneutic method inspired by Ricoeur [20, 21] and developed by Lindseth and Norberg [22] was applied. The method consists of three steps.

In the first step, *the naive reading*, the interviews were read several times in order to become familiar with the text and achieve an initial understanding. 

In the second step, *the structural analysis*, the texts were divided into meaning units in accordance with the aim of the study. The meaning units were then brought together and grouped into subthemes and themes. 

In the final phase, *the comprehensive understanding*, the text was read yet again, and the themes pertaining to what it means to recover after pancreaticoduodenectomy ad modum Whipple were reflected on. The interpretation was guided by the authors' preunderstanding based on their extensive experience of working in and teaching on the subject of surgery and surgical care, as well as their knowledge of previous research. In order to enhance credibility, the authors individually carried out the naive reading and structural analysis and subsequently analysed the text together to arrive at a comprehensive understanding. 

### 2.4. Ethical Considerations

According to Swedish law at the time this study was conducted, ethical approval was only required for research involving the risk of physical encroachment or aimed to affect the participants physically or psychologically and, therefore, not possible to obtain for this study [23], which was performed in accordance with the Declaration of Helsinki [24] and the ethical guidelines for nursing research valid at the time. The informants received an introductory letter where the background of the study was described. It also contained detailed information stating that participation was voluntary and that they were free to withdraw at any time. This information was given verbally before each interview. They were also assured of strict confidentiality and secure data storage.

## 3. Results

The naive understanding of the text revealed that, postoperatively, the informants had a strong will to recapture everyday life and were aware that they had to undergo a process of recovery. In the thematic structural analysis, the informants' experiences of recovery emerged as *recapturing everyday life, being healthy, and looking to the future* (Table 2). For many, support from family members, close friends, and health care providers was essential and enhanced their ability to adapt and manage. Their struggle and desire to regain their former capabilities and position in everyday life were present throughout the interview texts. The informants' main goal was to regain their former status in the social context and return to their former selves—a journey towards independence from caregivers and/or relatives.

### 3.1. Recapturing Everyday Life

After discharge from hospital, the informants were forced to confront everyday life on their own for the first time since surgery, without health care staff support. Postoperative symptom experiences at that time were lack of appetite, altered sense of taste, decreased physical functioning, and loose faeces. 

Food and drink were associated with negative experiences due to symptoms such as altered taste. Eating was no longer something positive or pleasant and merely considered necessary for the recovery process.

*“The most difficult part was coming home and finding that food was not tasty and that I was not hungry. I think it's fair to say that it was like being tired of food.”*

Due to difficulties with food intake, weight did not stabilise for a while. Bodily changes led to various emotional problems, for example, the experience that clothes did not fit or loathing themselves and their body.

*“I do not want to have close contact with other people. I realise that I do not like my own body at present. It was a shock that I should think that I was so repulsive.”*

The decrease in physical functioning was experienced as difficult to master and demanded a great deal of adaptation. The physical limitations made the informants more dependent on other people.

*“I was completely dependent on my husband for help, because I was very weak.”*

Initially, the symptoms had quite a significant impact on everyday life, leading to isolation or having to adapt to negative changes in bodily functioning. However, the informants mastered these experiences by using self-care and controlled the symptoms pharmacologically, for example, by means of analgesics and enzyme substitutes. Gradually, the symptoms abated. 

Recapturing everyday life involved planning in order to adapt to the new situation. This included planning meals and, in some cases, finding out the location of the nearest toilet. The unavoidable planning and daily changed routine were experienced as limiting. Before discharge, health care staff continuously provided the informants with attention and care, often creating a close relationship between them, which ceased after discharge. This was a shock for some informants, as they no longer had someone to rely on or with whom they could discuss their self-care experiences.

*“It may be that that's it. Now that I have been discharged they do not care about me as much as before. So now I'm discharged, written off somehow.”*

The informants underlined the importance of support from health care staff after discharge. It gave them an opportunity to discuss symptom management and self-care needs.

*“As soon as a problem arose, I phoned her. She always took the time and talked. If she wasn't in, she would phone back. It was nice to know that I could contact her.”*

Informants with enjoyable hobbies or who were still working were eager to regain their strength and impatient to return to their former lives. A gradual increase in the level of activity as well as social support helped many informants through this difficult transition, ensuring that they kept going in the right direction.

* “I have had tremendous support from family and friends as well as colleagues, but I suppose I am the sort of person who finds it easy to talk about it (my illness).”*

Difficulties were experienced by those with no close relatives or other support person. All the informants described the value of social support in the recovery process, not just with practical matters, but also in terms of encouragement.

*“It is tremendously important to me and means so much. I do not think I would have coped without that support because then I would have had to go into a home or something.”*

### 3.2. Being Healthy

The informants described themselves as healthy. The most important aspect of health was the ability to play an active part in everyday life and feel that they contributed both socially and professionally at work.

*“I have accompanied my son and worked a lot with him on different jobs, I find it great fun and being able to participate is equivalent to health.”*

Being healthy did not necessarily mean the absence of symptoms. In cases where the informants experienced very debilitating symptoms, they often coped by using successful symptom management.

*“Good health may not necessarily mean that I'm in top form but that I feel well, can manage my everyday life and think that living is great fun.”*

The importance of being needed at work and in their social network was emphasized. The initial experience of social isolation due to their symptoms made the informants angry, and they felt frustrated that readjustment took time. They never described themselves as sick, but instead stated that they experienced good health in view of their condition,which was associated with being independent.

*“Because I can, that I'm feeling well and can go out, can do various things without having to ask for help, just the fact that I'm able to do it gives you a kick, being able to manage thing.”*

### 3.3. Looking to the Future

All informants reflected on their future to some extent, but their perceptions varied. Some had a cautious short-term perspective and made the most of each day. Their strategy was not to dwell on the disease or a future relapse. They coped by adopting a positive way of thinking, focusing on good aspects of their present life and accepting the fact that the cancer might return.

*“Sometimes I think about what causes a relapse to occur, I mean how big is the risk of a new cancer in the pancreas, or some other form of cancer. But I have not bothered to ponder or read up about it as if it happens, it happens.” *

Older informants argued that having reached a considerable age, there was no point in speculating about life expectancy. However, all informants brooded and expressed uncertainty about the future. Sometimes, these uncertainties were related to information provided by health care staff or obtained by the informant herself/himself. Symptoms that reappeared gave rise to negative thoughts about the future and sometimes to frustration or anger. A contributory factor was a perception that health care staff had not provided straightforward and honest information. Sometimes informants searched for information about their condition, but had difficulty in understanding the content. When the informants were worried about the future, they frequently sought support, consolation, and motivation from their family members.

### 3.4. Interpreted Whole

Overall, the informants' view of the future was positive; they looked to the future with confidence, yet were aware of the risk of relapse. Improved health and recovery gradually contributed to a positive feeling and inspired them further in the healing process.

*“it was really fantastic that I recovered so quickly, which may explain why my self-confidence improved, not that it was lacking before but … strengthened self-confidence, strengthened belief [in one's ability] and all that…”*

## 4. Discussion

The main finding in this paper was that, despite experiencing several symptoms that affected everyday life, the informants perceived themselves as healthy. Hence, they managed the symptoms in order to regain their former selves and social lives. 

The participants in the present study reported experiences similar to patients recovering from breast, prostate, myeloma, colorectal, and other forms of cancer [25–28]. Common themes are the importance of support from family and friends, as well as dealing with symptoms related to treatment, for example, fatigue and diarrhoea. Another common theme is the fear of recurrent disease and death, which has also been reported in a Swedish context among patients recovering from upper gastrointestinal surgery [14]. The aforementioned Swedish study agrees well with the results of the present study in terms of postoperative symptoms, but not regarding the experience of being healthy and striving to regain a sense of social belonging. Previous studies have revealed that patients with pancreatic cancer exhibit high levels of psychosocial distress and depression [29–31]. However, this was not evident in the present study. 

Being healthy was described as being autonomous and part of a social context. Based on the analysis of the results, the experience of being in recovery after surgery for pancreatic or periampullary cancer was interpreted as recapturing everyday life, being healthy, and looking to the future. The informants appeared to be motivated and were proactive in self-care. The present self was characterised by recovery from various physical symptoms. The ideal self was described as their precancer self, which included being able to work and participate in family life as well as social activities. The main objective of their actions was interpreted as regaining their former status in the social context and returning to their former selves, which involved being independent of caregivers and relatives. 

The informants' desire for a quick recovery was a motivating factor. Obstacles that delayed the process could be the uncertainty of the cancer prognosis and the unpredictable future. The symptoms also constituted obstacles on the path to recovery and were mastered in different ways depending on severity and level of distress. Recovery after Whipple surgery for pancreatic or periampullary cancer meant being active and autonomous as well as needed in everyday life. 

This type of surgery has a long-term influence on a person's general state of health and can cause many postoperative symptoms that affect the recovery process. Lawler [32] described recovery as becoming independent and regaining control over one's bodily functions and body care. Parts of this description seem to be in line with the results of the present study, where striving for independence was part of the informants' progress towards recovery. 

According to Allvin et al. [32], the recovery process includes turning points or recovery indicators, defined as recovery trajectories. Critical junctures in the trajectory can be identified as improvements or setbacks in relation to expected outcome. Each trajectory corresponds with the progression towards recovery, and shifts are defined as recovery markers. In this study, a recovery trajectory passed at discharge when the informants had to manage by themselves, without being looked after by caregivers. However, they were not free from postoperative symptoms, nor were their functions or reestablished daily activities. Being in recovery concerned learning to manage symptoms. In the present study, regaining appetite and physical strength as well as mastering different self-care interventions represented small recovery markers. Being able to return to social activities and enjoying independence from caregivers seemed to be a marker of true recovery. This is in line with Allvin et al.'s [33] results, where patients and staff described postoperative recovery as “*A dynamic process in an endeavor to continue everyday life*” (p.3).

The informants' overall goal was to enjoy good health and be part of the social context. Their actions aimed at minimizing the gap between their initial condition before surgery and their actual condition during recovery. On the question of the meaning of health to the individual, the informants' response was regaining their former selves, that is, the way they were before the cancer, which they considered their ideal self. Being independent was another aspect of health. Despite the fact that the participants were in various phases of recovery, they assessed their health as good in view of the circumstances. The development of a new life plan includes self-assessment, self-determination, and self-realisation [34]. This implies having the will and capacity to develop a life plan, decide on realistic goals and a way of realising them. In the present study, looking to the future was a useful strategy for maintaining a sense of empowerment. The informants made an active choice not to focus on their present difficulties. This perception demonstrated that their efforts at self-realisation had been successful. 

The informants reflected on the cause, importance, and effects of the symptoms in their everyday lives and how they could be managed. During recovery, they consciously strived to improve their condition and master setbacks in order to continue the process. Other authors have described the physiological, emotional, social, and behavioural aspects of mastering [35, 36]. The extent to which the informants in the present study were able to use these abilities was dependent on their internal and external environments. The interaction between them is clearly visible in the informants' descriptions. The external environment, which mainly comprised family and relatives, was described as extremely important for formulating and achieving realistic goals and evaluating self-care. 

The informants' internal environment consisted of various types of emotion. Positive emotions such as hope and security sustained them through the recovery period, while self-evaluating emotions such as shame and guilt limited their repertoire of actions. For example, the informants expressed feelings of discomfort and shame about their own body as a result of bodily changes caused by weight loss. 

When the social context was unable to offer adequate support, health care staff had to provide confirmation. However, some informants did not obtain such confirmation, which made them feel abandoned. This was reported by Forsberg et al. [37], where liver transplant recipients described feelings of loneliness after discharge as well as of being abandoned by health care staff. Allvin et al. [33] also reported that patients felt abandoned after discharge, which could result in feeling dispirited and disengaged. 

The participants in the present study experienced similar symptoms to patients treated for abdominal or thoracic cancer. In addition, decreased physical functioning, symptoms related to eating and the eating experience, defecation problems, for example, diarrhoea and pain reported by our informants were similar to the experiences of patients treated for oesophagus, ventricle, breast, colon, lung, and prostate cancer [38–41]. The informants in the present study appeared to manage these symptoms by various strategies, which agree with previous findings among other cancer patients [15, 42, 43]. 

One unexpected and interesting finding was that the informants rarely dwelled on the reason for their present condition or on their prognosis. Nor did they reflect to any great extent on how they felt emotionally due to the fact that they had survived and were not dead or dying. The informants managed to cope with distressing symptoms, thoughts, and events. The ability to use coping strategies and be free of distressing symptoms fosters hope [44–46]. Many informants chose to seek support from their social network and not dwell on negative thoughts about the future and the risk of relapse, which Mattioli et al. [47] also recognized as a strategy for fostering hope. 

It is also important to reflect on the concept of denial. This is a common reaction among persons diagnosed with cancer and has been described by Vos et al. [48] as a normal state of mind in patients with lung cancer. Denial may have a positive or negative impact and is mediated by disease stage rather than type of tumour. When denial is related to improved psychological functioning, it represents an active strategy for coping with the situation [49]. However, when related to poor psychological functioning, denial involves passive and more negative strategies. The participants in the present study used active strategies such as seeking support from others to further improve their recovery. All participants had undergone curative treatment and experienced a positive yet realistic view of the situation and future, which could have affected denial. However, further studies with both inductive and deductive designs are required in order to draw general conclusions about the role and impact of denial during the recovery process after pancreatic surgery. 

### 4.1. Clinical Implications

An important question is what contribution nurses can make to patients' recovery process after discharge from hospital. We believe that it is possible for surgical nurses to support the patients' recovery even after discharge. Regular telephone followup could be of value in the recovery process, as it would allow the suffering caused by symptoms as well as setbacks to be addressed and might strengthen the patients' inner sense of security. The provision of adequate information adapted to the individual's needs, and ability to understand is of the utmost importance for postoperative recovery [33]. We believe that, as in all nursing, it is essential to adapt the care to the patient's experience of the situation and promote her/his self-reactivation during the recovery process. 

A characteristic of the patients in the present study was their strong will to live and their motivation to regain good health. Caregivers need to be responsive in order not to overlook the hope and driving force in each person. Our results demonstrate the importance of discussing the extent to which the patient has been successful in his/her recovery plan following discharge. The main focus of a telephone followup after discharge should be identifying recovery markers, supporting independence, and alleviating any suffering caused by impaired emotional well being or temporary setbacks. A recommendation is collaboration between different categories of health care professionals to address the problems experienced by patients after pancreaticoduodenectomy ad modum Whipple and to prepare a care plan. The findings highlight the importance of confirming and focusing on patients' resources and efforts to achieve good health during recovery. 

### 4.2. Methodological Limitations

The interpreted whole comprises the thematic analysis of the entire text, with focus on the meaning of patients' lived experience of recovery after pancreaticoduodenectomy ad modum Whipple due to a pancreatic or periampullary tumour. 

The informants represented a homogeneous group in terms of treatment but a heterogeneous group with regard to age, gender, and follow-up time. One limitation was that they were recruited from the same hospital, thus the findings reflect only one medical and nursing tradition. It is possible that the result could have been different had patients from other hospitals been included. However, the present trend is that this type of surgery is carried out in highly specialized centres. Our aim was not to evaluate the treatment but the experienced meaning of being in recovery from pancreaticoduodenectomy ad modum Whipple. It is important to consider the possibility that only patients who felt well agreed to participate while those who did not declined, which could have an impact on the result.

Trustworthiness is important in qualitative studies, which implies that the research process, as well as the final interpretation, must be transparent. Trustworthiness comprises credibility, transferability, dependability, and conformability [50]. To enhance the credibility of this study and prevent retrospective distortion or misinterpretation, the informants' statements were followed up by additional questions. The interviewer was able to enter deeply into the informants' experiences, due to being familiar with the context. Quotations were used to highlight the patients' voices, thus allowing the reader to decide whether or not our descriptions and interpretations are reasonable, as well as to reflect on the meaning of the statements. With regard to dependability and conformability, the full interview text was read and reflected on by all the authors, and only statements relevant to the aim were incorporated into the final analysis. We included data that contained both typical and atypical elements. Conformability/neutrality, which is the equivalent of reliability in quantitative studies, was achieved by our description of the different steps of the analytical process and how the various themes in the structural analysis were created on the basis of quotations. Transferability was ensured by the fact that the transcribed text was rich and extensive,making it possible to transfer the findings to other patients undergoing pancreaticoduodenectomy ad modum Whipple. The authors agreed that the final interpretation was the most reasonable, although alternative interpretations were considered. 

Informants included in this study were all treated by means of surgery between 2006 and 2008. However, as the surgical procedure and nursing care for these patients are still the same today, the results are applicable to the current care of this patient group. 

## 5. Conclusion

Our study indicates that patients recovering from pancreaticoduodenectomy ad modum Whipple due to a pancreatic or periampullary tumour experience health and express a positive view of the future, despite postoperative symptoms, which they manage by different strategies. This health process is driven by a will to belong to and be part of a social context. These patients use goal-oriented actions to recapture everyday life, be healthy, and look to the future. Nurses working with such patients should adopt a person-centred approach focusing on their perspectives, participation, and possibilities. The results presented in this paper demonstrate that despite being burdened by postoperative symptoms, the patients can experience health and look to the future.

## Figures and Tables

**Table 1:** Demographic data (*n* = 13).

Age (mean, range)	66, 54–76
Sex (male/female)	4/9
Civil status (married/single/widower)	10/2/1
Employment status (full time/part time/retired/sick leave)	0/3/8/2
Months since discharge (mean, range)	6, 1–15
Tumour site (pancreas, duodenum, ampulla vateri)	9, 3, 1

**Table 2:** Thematic structural analysis of the patients' experiences of being in recovery after pancreaticoduodenectomy ad modum Whipple (*n* = 13).

Subtheme	Main theme
Living with and managing symptoms	
Mastering self-care	
Planning daily life	Recapturing everyday life
Receiving support from health care staff	
Being part of a social context	

Feeling well as opposed to ill	
Being active and independent	Being healthy
Feeling needed and included	
Returning to an active life	

Pondering about the future	
Thinking positively	Looking to the future
Being in a state of uncertainty	
